# Chronic Consumption of Farmed Salmon Containing Persistent Organic Pollutants Causes Insulin Resistance and Obesity in Mice

**DOI:** 10.1371/journal.pone.0025170

**Published:** 2011-09-23

**Authors:** Mohammad Madani Ibrahim, Even Fjære, Erik-Jan Lock, Danielle Naville, Heidi Amlund, Emmanuelle Meugnier, Brigitte Le Magueresse Battistoni, Livar Frøyland, Lise Madsen, Niels Jessen, Sten Lund, Hubert Vidal, Jérôme Ruzzin

**Affiliations:** 1 National Institute of Nutrition and Seafood Research, Bergen, Norway; 2 Institute of Biomedicine, University of Bergen, Bergen, Norway; 3 Department of Biology, University of Copenhagen, Copenhagen, Denmark; 4 INSERM U-1060, INRA U-1235, CarMeN Laboratory, Lyon1 University, Oullins, France; 5 Department of Clinical Pharmacology, Aarhus University Hospital, Aarhus, Denmark; 6 Department of Internal Medicine and Diabetes and Institute of Experimental Clinical Research, Aarhus University Hospital, Aarhus, Denmark; 7 Department of Biology, University of Bergen, Bergen, Norway; University of Padova, Italy

## Abstract

**Background:**

Dietary interventions are critical in the prevention of metabolic diseases. Yet, the effects of fatty fish consumption on type 2 diabetes remain unclear. The aim of this study was to investigate whether a diet containing farmed salmon prevents or contributes to insulin resistance in mice.

**Methodology/Principal Findings:**

Adult male C57BL/6J mice were fed control diet (C), a very high-fat diet without or with farmed Atlantic salmon fillet (VHF and VHF/S, respectively), and Western diet without or with farmed Atlantic salmon fillet (WD and WD/S, respectively). Other mice were fed VHF containing farmed salmon fillet with reduced concentrations of persistent organic pollutants (VHF/S_-POPs_). We assessed body weight gain, fat mass, insulin sensitivity, glucose tolerance, *ex vivo* muscle glucose uptake, performed histology and immunohistochemistry analysis, and investigated gene and protein expression. In comparison with animals fed VHF and WD, consumption of both VHF/S and WD/S exaggerated insulin resistance, visceral obesity, and glucose intolerance. In addition, the ability of insulin to stimulate Akt phosphorylation and muscle glucose uptake was impaired in mice fed farmed salmon. Relative to VHF/S-fed mice, animals fed VHF/S_-POPs_ had less body burdens of POPs, accumulated less visceral fat, and had reduced mRNA levels of *TNFα* as well as macrophage infiltration in adipose tissue. VHF/S_-POPs_-fed mice further exhibited better insulin sensitivity and glucose tolerance than mice fed VHF/S.

**Conclusions/Significance:**

Our data indicate that intake of farmed salmon fillet contributes to several metabolic disorders linked to type 2 diabetes and obesity, and suggest a role of POPs in these deleterious effects. Overall, these findings may participate to improve nutritional strategies for the prevention and therapy of insulin resistance.

## Introduction

Insulin resistance is a critical defect in the pathogenesis of obesity and type 2 diabetes. Alarmingly, the incidence of these diseases has exploded worldwide, and is now reaching epidemic proportions. In the United States, over 25% of adults are affected by metabolic abnormalities associated with insulin resistance [1]. Similar situation has been documented in the European Union where over half the adult population is estimated to be overweight or obese [2]. In 20–25 years, 600 million people are expected to be obese, and ∼370 million persons will develop diabetes [3].

Dietary strategies have attracted extensive interest in our search to halt and turn back the threat of insulin resistance-associated metabolic diseases. However, the effectiveness of dietary approaches to prevent and treat metabolic disorders have remained challenging [4], [5]. During the last years, considerable focus has been directed toward fatty fish and the influence of fish intake on type 2 diabetes and other metabolic diseases remains ambiguous. On one hand, very long-chain n-3 polyunsaturated fatty acids (LC n-3 PUFA), primarily eicosapentaenoic acid (EPA) and docosahexanoic acid (DHA), and fish protein have been documented to protect against insulin resistance and cardiovascular disease [6]–[11]. Furthermore, fish may also provide essential micronutrients and bioactive compounds with potential health benefits [12], [13]. On the other hand, recent studies reported that fish consumption had no beneficial effects on the risk of type 2 diabetes and rather, enhanced the incidence of the disease [14]–[16]. We have, in addition, demonstrated that the presence of environmental contaminants, like persistent organic pollutants (POPs), in fish oil may counteract the benefits of LC n-3 PUFA [17].

There are currently no published experimental data regarding the metabolic impacts associated with intake of fatty fish as a whole -combined effects of fish oil and fish meat-. In the present study, we investigated whether farmed salmon fillet could protect against the development of insulin resistance induced by a very high-fat diet (VHF) or Western diet (WD) in mice. Furthermore, we assessed whether the outcomes of salmon fillet intake could be modulated by the presence of POPs.

## Methods

### Ethics statement

All experimental protocols were approved by the Norwegian State Board of Biological Experiments with Living Animals (authorization number: 1296, 1588, 2285).

### Animal studies

Eight-week-old male C57BL/6J mice (Taconic, Ry, Denmark) were housed on a 12 h light/dark cycle, with free access to food and tap water. Mice were divided into weight matched groups and fed either control (C; 2018 Teklad Global, Harlan Laboratories, The Netherlands), VHF, VHF containing farmed salmon fillet (VHF/S) or VHF containing farmed salmon fillet with reduced POP concentrations (VHF/S_-POPs_) for 8 weeks. Level of protein in the purified diets was adjusted to an isonitrogenous basis at the expense of carbohydrates and corn oil was adjusted according to lipids present in salmon fillet so that total dietary fatty acid concentrations were similar in all purified diets (Table S1). Accordingly, experimental diets contained (in ∼percentage of calories): 16% protein (casein with no added amino acids), 72% fat (50% lard and 50% corn oil), and 12% carbohydrate (sucrose) for VHF; 16% protein (salmon), 72% fat (50% lard, 19% corn oil, and 31% salmon oil from salmon fillet), and 12% carbohydrate (sucrose) for both VHF/S and VHF/S_-POPs_. The energy content of purified diets was measured in a bomb Parr calorimeter 6300 (Parr Instruments, Moilne, IL, USA) and was found to be isocaloric (∼26.5 kJ/g).

In another set of experiments, mice were fed either chow low-fat diet (C; D12450B Research Diet, Taconic, Gentofte, Denmark), WD, or WD containing farmed salmon fillet (WD/S) for 6 weeks. Purified diets contained 19% protein (casein with no added amino acids), 29% fat (corn oil), and 52% carbohydrate (88% sucrose and 12% cornstarch) for WD; 19% protein (salmon), 29% fat (salmon oil from salmon fillet), and 52% carbohydrate (88% sucrose and 12% cornstarch) for WD/S. Energy content of both WD and WD/S was ∼20.5 kJ/g.

All purified diets were supplemented with cellulose, choline bitartrate, AIN vitamin mixture 76, and AIN mineral mixture 76 as described previously [17]. Once made, experimental purified diets were screened for POP concentrations (Table S2).

Casein (no added amino acids), lard, cellulose, choline bitartrate, AIN vitamin mixture 76, and AIN mineral mixture 76 were purchased from MP Biomedicals (Illkrich, France), corn oil was from Landlord (Reitangruppen, Denmark), cornstarch was from Primeal (Peaugres, France), and sucrose was from Dan Sukker (Oslo, Norway).

### Salmon fillets

Commercial farmed Atlantic salmon fillets were bought from an international fish retailer (Leroy Seafood Group, Bergen, Norway). To reduce the presence of POPs in farmed salmon fillet, we tailored Atlantic salmon by feeding them a standard fish feed in which the fish oil used was purified rather than crude; crude fish oil being the principal source of POPs in fish feed [18]. Through this approach, we managed to produce farmed salmon fillet with ∼50% lower POP concentrations compared with commercial farmed salmon fillet (Table S3). After 8 weeks, Atlantic salmon (1137±215 g, *n* = 36) were filleted. Both commercial and tailored farmed salmon fillets were then freeze dried and homogenised. Before being used for animal studies, homogenised salmon fillets were analyzed for crude protein level with a Leco FP-528 nitrogen analyzer (Leco Corp, St. Joseph, MI, USA), amino acid levels were assessed with Acquity Ultraperformance LC (Waters, Milford, Massachusetts, USA), lipid content and fatty acid composition were analysed as described previously [17], [19]. Characteristics of salmon fillets are shown in Table S3.

### POP analysis

Concentrations of organochlorine pesticides, dioxins, furans, dioxin-like polychlorinated biphenyls (dl-PCBs), and 7 polychlorinated biphenyls (7PCBs; PCB28,52,101,138,153,180) were measured as described previously [17].

### Tissue harvest

Overnight fasted animals were anaesthetized with isoflurane. Organs were dissected, weighed, dipped in liquid nitrogen, and stored at −80°C until further analysis. Other tissues were fixed for histological investigations.

### Assessment of triacylglycerol (TAG) levels

Concentrations of TAG were determined in gastrocnemius muscle and liver (left lobe) samples of overnight-fasted mice as described previously [17].

### Assessment of fat absorption

Three weeks before the end of experiments, feces present in cages were collected over a period of 7 days. After collection, feces were dried, weighed, and total lipid was extracted from feces by homogenization in chloroform:methanol, (2∶1, v/v) with 19∶0 methyl ester as internal standard as reported previously [20].

### Histology and immunohistochemistry

Small pieces of epididymal or liver (left lobe) fat were fixed overnight at 4°C by immersion in phosphate buffered 4% paraformaldehyde. Next, samples were dehydrated and embedded in paraffin. Hematoxylin and eosin (H&E) staining was performed on 5 µm thick sections to assess morphology. Other 5 µm sections were deparafinized, rehydrated and endogenous peroxide was inactivated by 3% hydrogen peroxide. To reduce non-specific staining the sections were incubated in heat-inactivated normal goat serum (10%, 10 min). Sections were then incubated overnight at 4°C with rat anti-mouse F4/80 (1∶500; AbD Serotec, Oxford, England), and then incubated ith rat anti-goat-IgG (1∶250; AbD Serotec, Oxford, England) conjugated with horseradish peroxidase for 2 h. Specific binding was visualised using diaminobenzidine.

### Plasma insulin and blood glucose

We collected blood from the tail vein of conscious animals one week before the end of feeding trial. Plasma insulin concentrations were assessed by ELISA (Mouse Insulin ultrasensitive ELISA, DRG, Marburg, Germany). Blood glucose levels were measured with a glucometer (Ascensia Contour, Bayer Healthcare, Oslo, Norway).

### Insulin and glucose tolerance tests

Insulin and glucose tolerance tests were performed in fed and 5 h fasted mice, respectively. At week 7 and 5 for VHF and WD trials, respectively, mice were injected i.p. with human insulin (Actrapid, Novo Nordisk, Bagsværg, Denmark; 1.0 U/kg body weight) or glucose (1.0 mg/g body weight), and blood glucose was measured prior to injection and at 15, 30, 60, and 90 min after injection.

### Assessment of insulin secretion

One week before the end of feeding trial, 5 h fasted mice were injected i.p. with 2 mg glucose/g body weight and plasma insulin measured at 0 and 15 min post-injection.

### Insulin signaling

*In vivo* insulin stimulation was performed as described previously [21], with minor modifications. Overnight fasted animals were injected i.p. with saline or human insulin (20 U/kg body weight), and 10 min later, whole gastrocnemius muscles (both white and red parts) were harvested, frozen in liquid nitrogen, and stored at −80°C. Frozen muscle samples were weighed, homogenised and Akt/phospho-Akt (Ser^473^) was measured by immunoblotting as described previously [22]. Each sample was run at least three times. Antibody binding was detected by enhanced chemiluminescence (ECL; Amersham Pharmacia, Biotech, Buckinghamshire, UK). For semi-quantification of immunoblots, chemiluminescence imaging was assessed by G:Box system with Gene Snap software, and images were analyzed by Gene Tool software (Syngene, Cambridge, England). Primary and secondary antibodies were from Cell Signaling (Beverly, MA, USA).

### *Ex vivo* muscle glucose uptake

Glucose uptake was assessed in intact soleus muscle, a small thin slow-twitch muscle, incubated without or with insulin (60 nmol/l) as described previously [22].

### Adipocyte differentiation

3T3-L1 preadipocytes were grown to confluence in standard high glucose Dulbecco's modified Eagle's medium (DMEM; PAA, Les Mureaux, France) supplemented with 10% bovine serum (PAA) at 37°C in a humidified atmosphere of 5% CO_2_. Two days after reaching confluence, differentiation was initiated by incubation in DMEM supplemented with 10% fetal bovine serum, FBS (PAA), 10 µg/ml insulin (Sigma-Aldrich, Saint-Quentin Fallavier, France), 50 nM cortisone (Sigma-Aldrich) to avoid maximal differentiation [23], and 0.5 mM isobutylmethylxanthine (Sigma Aldrich). After two days, the medium was removed and the cells were cultured for three additional days in DMEM containing 10% FBS and insulin supplemented or not with different concentrations of pp′-dichlorodiphenyldichloroethylene (pp′-DDE; Sigma Aldrich). The medium was then removed and the cells were maintained in 10% FBS medium alone without pp′-DDE for two days. Lipid accumulation was quantified using Oil Red O staining. Oil red O was dissolved in isopropanol overnight at a concentration of 0.35% followed by filtration, dilution in water to a final concentration of 0.2%. Differentiated adipocytes were washed with PBS and fixed in 10% formalin for 60 min. After washing, cells were stained with Oil Red O for 10 min at room temperature. Following washes, cells were dried and Oil Red O was eluted by 100% isopropanol and absorbance was measured at 500 nM.

### Quantification of mRNAs using real-time PCR

Total RNA was prepared from epididymal fat, and real-time PCR assays were performed as described previously (17). Briefly, first-strand complementary DNA's (cDNAs) were first synthesized from 250 ng of total RNA in the presence of 100 units of Superscript II (Invitrogen, Eragny, France) using a mixture of both random hexamers and oligo (dt) primers. The real-time PCR was performed with a Rotorgene Q (Qiagen, Courtaboeuf, France) in a final volume of 20 µl containing 5 µl of a 60-fold dilution of the RT reaction medium, and 15 µl of reaction buffer from the ABsolute QPCR SYBR Green Mix (Thermo Scientific). For quantification, a standard curve was systematically generated with 6 different amounts (150 to 30,000 molecules/tubes) of purified target cDNA cloned in the pGEM plasma (Promega, Charbonnières, France). Each assay was performed in duplicate. Data were normalized using TATA Box Binding protein (TBP) mRNA levels measured in each sample by real-time PCR as an internal standard and then expressed as arbitrary unit (AU). Primer sequences are available upon request (hubert.vidal@univ-lyon1.fr).

### Statistics

Results are expressed as mean ± SE. Differences between groups were examined for statistical significance using analysis of variance (ANOVA) with the least-square difference (LSD) post hoc test. Repeated measures ANOVA was used on data obtained from tolerance tests. When appropriate, statistical significance was determined using Student's t test. Differences were considered significant at *p*<0.05.

## Results

### Intake of farmed salmon fillet causes visceral obesity and chronic-low grade inflammation in adipose tissue of mice fed VHF

**Figure 1:**
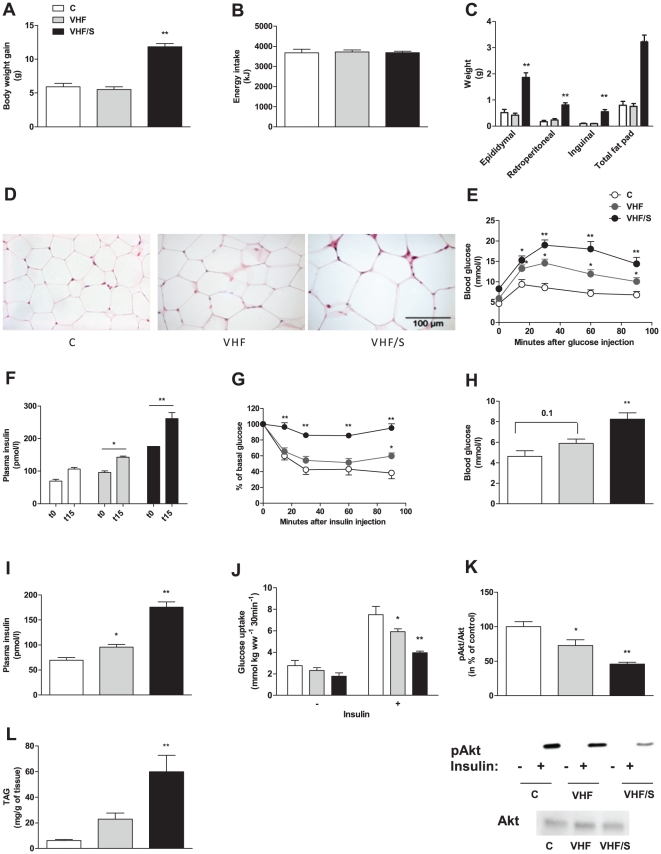
Mice fed VHF/S developed obesity and insulin resistance. In two separate studies, mice fed C (*n* = 14), VHF (*n* = 43) or VHF/S diet (*n* = 38) were monitored for 8 weeks and assayed for various metabolic parameters. (**A**) Body weight gain (14–43 mice per group). (**B**) Energy intake (14–43 mice per group). (**C**) Quantification of adipose tissue. Total fat pad includes epididymal, retroperitoneal and inguinal fat pad (7–16 mice per group). (**D**) H&E staining showing representative morphology of adipocyte in epididymal fat of animals (4–5 mice per group). (**E**) Glucose tolerance test. Glucose was injected and blood glucose was assessed at indicated time points (7–13 mice per group). (**F**) Glucose-stimulated insulin release. Plasma insulin levels were measured before and 15 min after injection of glucose in mice (8–12 mice per group). (**G**) Insulin tolerance test. Random-fed mice were injected with insulin and blood glucose assessed at indicated time points (7–13 mice per group). (**H**) Blood glucose (4–7 mice per group) (**I)** Plasma insulin (4–6 mice per group). (**J**) Muscle glucose uptake. *Ex vivo* soleus muscles were incubated without or with insulin and glucose uptake assessed (7–12 mice per group). (**K**) *In vivo* insulin signaling. Overnight fasted animals (n = 4–5 per group) were injected with insulin or saline and expression of Akt and pAkt in gastrocnemius muscles was assessed. Graphic depicts densitometric analysis of normalization of pAkt/Akt protein. Representative western blots of muscle lysates are shown for phosphorylated Akt (Ser^473^) without or with insulin stimulation, and for total Akt expression after saline injection. Western blot analyses were repeated at least three times. (**L**) Triacylglyceride (TAG) concentrations in gastrocnemius muscles (6–12 mice per group). *p<0.05 vs. C. **p<0.03 vs. VHF.

VHF feeding was reported to develop a diabetic phenotype before the onset of obesity in mice [24]. We took advantage of this dietary model and investigated the impacts of farmed salmon fillet intake in VHF-fed mice. After 8 weeks, mice fed VHF/S gained about two times more weight than other animals (Figure 1A) despite similar energy intake (Figure 1B). Interestingly, VHF/S-fed mice were characterized by enhanced fat absorption as demonstrated by reduced fat excretion in feces (Figure S1), a mechanism that likely participated to increase body weight gain of these animals. In association with their increased body weight, animals fed VHF/S exhibited increased visceral fat (Figure 1C), which was associated with a prominent increased of adipocyte size in epididymal fat pad (Figure 1D). Furthermore, the expression of *Mac2-a*, a galactose-binding lectin expressed by activated macrophages, was increased by about 13-fold in epididymal fat of animals fed VHF/S compared with VHF (Table 1), thereby suggesting macrophage infiltration in adipose tissue of animals exposed to farmed salmon fillet. Because activated macrophages may release inflammatory molecules, we next measured expression of *IL-6, iNOS*, and *TNFα*. In white adipose tissue of mice fed VHF/S, mRNA levels of *TNFα* and *iNOS* were up-regulated by about 5- and 3-fold, respectively, compared with VHF-fed mice whereas *IL-6* expression was unchanged (Table 1). In concert, these results indicated that farmed salmon feeding robustly increases body weight and fat mass, and induces chronic-low grade inflammation.

**Table 1:** Expression of inflammatory markers in epididymal fat of animals.

Inflammatory genes (arbitrary units)	VHF	VHF/S
*Mac2-a*	2.6±0.4	34.4±8.0
*IL-6*	7.4±3.5	6.5±0.9
*TNFα*	5.8±1.6	35.3±7.6
*iNOS*	3.8±0.5	11.9±1.8

### Intake of farmed salmon fillet exaggerates insulin resistance and glucose intolerance in mice fed VHF

We next examined glucose homeostasis of animals by challenging them to a glucose tolerance test. In comparison with C-fed mice, mice fed VHF had higher blood glucose levels at all time points following the glucose load (Figure 1E), thereby confirming the diabetic phenotype of VHF-fed mice despite the absence of obesity. Importantly, dysregulation of whole-body glucose homeostasis was worsen in mice fed VHF/S (Figure 1E), and was associated with increased insulin production in response to glucose challenge (Figure 1F). VHF/S-fed mice also exhibited a reduced response to glucose clearance following insulin load relative to VHF- and C-fed mice (Figure 1G); all features demonstrating a state of insulin resistance. Accordingly, mice fed VHF/S had increased blood glucose (Figure 1H) and plasma insulin (Figure 1I) relative to other animals. Because skeletal muscle is a predominant peripheral site of insulin-dependent glucose disposal [25], we further investigated insulin-stimulated glucose uptake in soleus muscles of animals. In the absence of insulin, basal muscle glucose uptake was similar in all experimental groups (Figure 1J). However, the ability of insulin to stimulate glucose uptake was reduced in muscles of mice fed VHF (-22%) and VHF/S (-47%) compared with C-fed mice (Figure 1J). In accordance with these results, we found that insulin-stimulated phosphorylation of Akt, a key insulin signaling protein, was decreased in skeletal muscles of both VHF and VHF/S-fed mice relative to animals fed control diet (Figure 1K). Loss of insulin action in skeletal muscles was associated with enhanced muscle TAG accumulation (Figure 1L), which is consistent with previous report [26]. Furthermore, the development of hepatic steatosis, which is a severe manifestation of metabolic syndrome, was also exacerbated in mice fed farmed salmon fillet (Figure S2). By using F4/80 staining, no mature macrophage infiltration was found in livers of VHF/S-fed mice (data not shown). Altogether, these data demonstrated that intake of farmed salmon fillet caused visceral obesity and accelerated the development of insulin resistance induced by VHF feeding.

### Intake of farmed salmon fillet contributes to visceral obesity and insulin resistance mice fed WD

**Figure 2:**
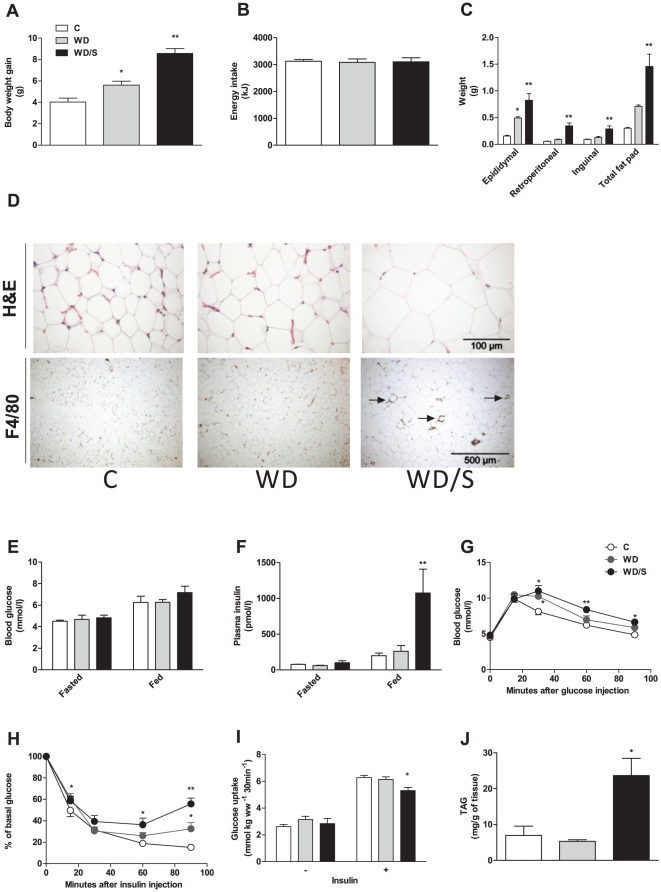
Intake of WD/S exacerbated obesity and insulin resistance. In two separate studies, mice fed C (*n* = 8), WD (*n* = 15) or WD/S (*n* = 15) diet were monitored for 6 weeks. (**A**) Body weight gain (8–15 mice per group). (**G**) Energy intake (8–15 mice per group). (**C**) Quantification of adipose tissue. Total fat pad includes epididymal, retroperitoneal and inguinal fat pad (4–6 mice per group). (**D**) Representative H&E staining (upper panel) and immunohistochemical detection of the macrophage-specific antibody F4/80 (lower panel) in epididymal fat (4–5 mice per group). Note the abundance of macrophages (arrows) surrounding adipocytes, crown-like structures, in epididymal fat of WD/S-fed animals. (**E**) Blood glucose and (**F**) plasma insulin was determined in random-fed and fasted mice (4–7 mice per group). (**G**) Glucose tolerance test. Glucose tolerance test was performed by injection of glucose in fasted mice and blood glucose was assessed at indicated time points (4–7 mice per group). (**H**) Insulin tolerance test. Insulin tolerance test was performed by injection of insulin in random-fed mice and blood glucose was assessed at indicated time points (4–7 mice per group). (**I**) Muscle glucose uptake. Glucose uptake was assessed in *ex vivo* soleus muscles incubated without or with insulin (4–6 mice per group). (**J**) TAG concentrations in gastrocnemius muscles (4-6 mice per group). *p<0.05 vs. C. **p<0.04 compared with WD.

To confirm the ability of farmed salmon fillet to induce insulin resistance and visceral obesity in a more conventional experimental diet inducing both insulin resistance and obesity, we challenged animals with high-fat/high-carbohydrate diet; WD. After 6 weeks, mice fed WD/S had higher body weight gain than mice fed WD and C (Figure 2A) despite similar energy intake (Figure 2B). As observed under VHF feeding, the percentage of fat excreted in feces was largely reduced in mice fed WD/S relative to WD (Figure S3), thereby suggesting increased intestinal fat absorption in WD/S-fed mice. Consistent with enhanced body weight, mice fed WD/S were characterized by an overgrowth of adipose tissue, including epididymal, retroperitoneal and inguinal fat pad (Figure 2C), and was associated with adipocyte hypertrophy and macrophage infiltration (Figure 2D). Next, we assessed glucose tolerance and insulin sensitivity of animals. In fasted state, there were no differences in blood glucose (Figure 2E) and plasma insulin concentrations (Figure 2F). However, in fed condition, a mild increase of blood glucose (Figure 2E) and dramatic increase in plasma insulin levels (Figure 2F) was found in mice challenged with WD/S, suggesting a whole-body insulin resistance state. In accordance, both glucose and insulin tolerance tests revealed that animals fed WD/S had impaired glucose tolerance (Figure 2G) and systemic insulin resistance (Figure 2H). Furthermore, insulin-stimulated glucose uptake in skeletal muscles was significantly reduced in WD/S-fed mice (Figure 2I), and correlated with increased muscle TAG storage (Figure 2J). Elevated TAG concentrations in liver of animals fed WD/S were also found (Figure S2). Taken together, these results demonstrated that mice fed WD/S, similarly to animals fed VHF/S, developed serious disorders linked to type 2 diabetes and obesity.

### Potential implications of POPs in the deleterious metabolic effects associated with farmed salmon intake

**Figure 3:**
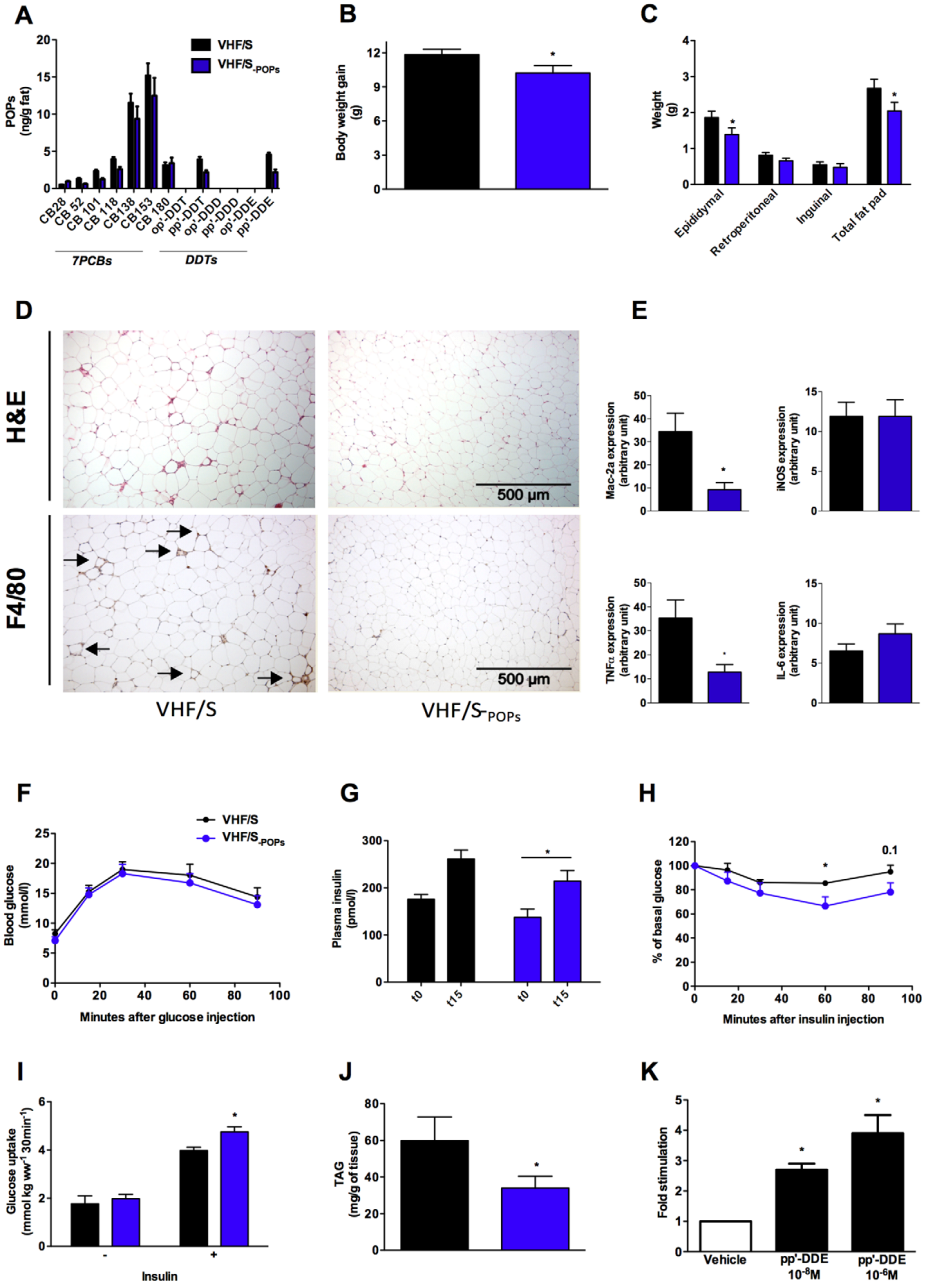
POPs modulated the outcomes of farmed salmon intake. In three separate studies, mice fed VHF/S (*n* = 31) and VHF/S_-POPs_ (*n* = 25) for 8 weeks were screened for insulin resistance-induced metabolic disorders. (**A**) Concentrations of 7PCBs and DDTs in epididymal fat of animals (5 mice per group). (**B**) Body weight gain (25–31 mice per group). (**C**) Quantification of adipose tissue. Total fat pad includes epididymal, retroperitoneal and inguinal fat pad (8–11 mice per group). (**D**) Representative H&E staining (upper panel) and immunohistochemical detection of the macrophage-specific antibody F4/80 (lower panel) in epididymal fat (4–5 mice per group). Note the important infiltration of macrophages in epididymal fat (arrows) of mice fed VHF/S compared with VHF/S_-POPs_ . (**E**) Real-time PCR determination of mRNA expression of *Mac-2a, iNOS, TNFα and IL-6* in epididymal fat (5 mice per group). (**F**) Glucose tolerance test. Mice were injected with glucose and blood glucose assessed at indicated time points (8–13 mice per group). (**G**) Glucose-stimulated insulin release. Plasma insulin levels were measured before and 15 min after glucose injection (6–10 mice per group). (**H**) Insulin tolerance test. Random-fed mice were injected with insulin and blood glucose assessed at indicated time points (8–16 mice per group). (**I**) Muscle glucose uptake. *Ex vivo* soleus muscles were incubated without or with insulin, and glucose uptake assessed (6–12 mice per group). (**J**) TAG concentrations in gastrocnemius muscles (6–10 mice per group). (**K**) 3T3-L1 preadipocytes were treated with a weak differentiation cocktail containing cortisone and exposed to the organochlorine pesticide pp′-DDE. Graphic shows fold stimulation of lipid accumulation quantified by Oil red O staining. Results are expressed relative to vehicle-treated cells for three independent experiments. *p<0.05 vs. VHF/S or vehicle-treated cells.

We have previously reported that POPs may counteract the benefits of salmon oil and impair insulin action in both *in vivo* and *in vitro* models [17]. To determine whether the presence of POPs modulates the outcomes of farmed salmon fillet intake, we compared the metabolic profile of mice challenged with VHF/S to those fed VHF/S_-POPs_ (Table S1 and S2). In epididymal fat, mice fed VHF/S_-POPs_ had about 20% and 50% lower concentrations of 7PCBs and dichlorodiphenyltrichloroethanes (DDTs), respectively, than animals fed VHF/S (Figure 3A), thereby indicating that mice fed VHF/S_-POPs_ had reduced body burdens of POPs. Interestingly, mice fed VHF/S_-POPs_ had reduced body weight gain (Figure 3B) and visceral fat compared with animals fed VHF/S (Figure 3C). This decrease in adipocity was correlated with reduced adipocyte size and macrophage infiltration as revealed by H&E and F4/80 staining (Figure 3D), as well as reduced *Mac2-a* expression (Figure 3E). In addition, mRNA levels of *TNFα*, but not *IL-6* and *iNOS*, in white adipose tissue of mice fed VHF/S_-POPs_ were significantly down-regulated compared with VHF/S-fed mice (Figure 3E). There were no significant differences in energy intake and fat absorption between groups (data not shown).

Further, we investigated whether mice fed VHF/S_-POPs_ had comparable glucose intolerance and insulin resistance to animals fed VHF/S. In fasted state, blood glucose tended to decrease in VHF/S_-POPs_ relative to VHF/S-fed animals (7.1±0.6 vs. 8.3±0.6 mmol/l, respectively), whereas plasma insulin concentrations were significantly reduced in mice challenged with VHF/S_-POPs_ compared with VHF/S (137.5±17.4 vs. 175.7±10.44 pmol/l, respectively). Following glucose injection, blood glucose response was similar between mice fed VHF/S and VHF/S_-POPs_ (Figure 3F) whereas glucose-stimulated insulin production was reduced in mice fed VHF/S_-POPs_ (Figure 3G). Furthermore, mice fed VHF/S_-POPs_ had a tendency to better whole-body insulin sensitivity following insulin load (Figure 3H). To investigate this further, we measured *ex vivo* muscle glucose uptake. Consistent with enhanced insulin action, animals consuming VHF/S_-POPs_ had improved insulin-stimulated glucose uptake in soleus (Figure 3I), which was accompanied by reduced muscle TAG accumulation (Figure 3J), and tended to have less hepatic TAG concentration (Figure S2). Overall, these results highlight that POPs may affect the metabolic impacts induced by farmed salmon fillet intake. To further confirm a causal role of POPs in metabolic disorders, especially in adipose tissue related disorders, we investigated the ability of pp′-DDE, the DDT congener most present in salmon fillet, to stimulate lipid accumulation in 3T3-L1 cells. In this murine cell line, exposure to pp′-DDE was found to enhance lipid accumulation compared with vehicle-treated cells (Figure 3K). This finding highlights the ability of this organochlorine pesticide to regulate adipose tissue biology, and extends our previous observations that exposure to DDT mixture containing pp′-DDE impairs insulin action and glucose uptake in 3T3-L1 cells [17].

## Discussion

In the present study, we reported that inclusion of farmed salmon fillet in two different experimental diets, VHF and WD, contributed to insulin resistance and obesity in mice. Furthermore, our data suggested that the presence of POPs may participate to the deleterious metabolic outcomes associated with farmed salmon fillet intake.

The impacts of fatty fish consumption on chronic and metabolic diseases remain unclear. Intake of fish and LC n-3 PUFA was reported to either ameliorate [9], [27], [28], have no effect [29]-[33], or impair [34]–[39] glycemic control and insulin sensitivity. Furthermore, intake of LC n-3 PUFA was found to enhance the risk of diabetes in older women [40]. This finding was recently supported by three prospective follow-up studies. In the United States, after a ∼3 million person-years of follow up, LC n-3 PUFA and fish consumption was reported to enhance the incidence of type 2 diabetes [15]. In accordance, a ∼12 years follow-up of American women highlighted a positive association between fatty fish and LC n-3 PUFA consumption and type 2 diabetes [16]. In Europe, a 15 years of follow-up investigation revealed a positive association for fish intake and diabetes risk in Dutch adults [14]. Consistent with these findings, we provide here the first experimental evidence that diet containing fatty fish -farmed Atlantic salmon fillet- may cause, in rodents, several disorders associated with type 2 diabetes and obesity.

Identification of mechanisms behind the deleterious effects of farmed salmon fillet remains challenging because fatty fish represents a complex food matrix. There is emerging evidence suggesting that environmental contaminants like POPs may modulate the health effects of seafood consumption. Lipophilic POPs are mainly present in the lipid fraction of fish and their concentrations are therefore expected to be higher in fatty fish than in lean fish. Interestingly, delipidated salmon protein hydrolysate and cod fillet (<0.2% lipid) were found to prevent the development of insulin resistance in obese rats fed high-fat diet [10], [12], thereby highlighting that fish protein, with very low concentrations of lipids and POPs, may have beneficial effects. In addition, we have reported that consumption of salmon oil with very low POP levels protected against insulin resistance and visceral obesity in rats, whereas intake of similar oil containing environmental levels of POPs contributed to the development of metabolic disorders [17]. In the present study, we further demonstrated that glucose and lipid homeostasis was less disturbed in mice fed VHF/S_-POPs_ relative to mice fed VHF/S, thereby suggesting that POPs may affect the outcomes of fatty fish, i.e. both fish protein and fish oil. In addition, we found that pp′-DDE interacted with adipose tissue biology, which is in line with the established endocrine disruptor features of xenobiotics [41]–[43]. In concert, these findings suggest that inconsistency of studies investigating the impacts of fish products and LC n-3 PUFA on metabolic diseases may, in part, be ascribed to the different concentrations of POPs present in fish and marine oils.

Although people are regularly advised to consume healthy and varied food products as well as to exercise, the global prevalence of chronic metabolic diseases continues to increase at alarming rates. This worrying situation clearly reflects the urgency to optimize current dietary and lifestyle strategies to fight insulin resistance and obesity. During the last years, several studies reported that diabetes is associated with increased body burdens of POPs [44]–[46], and a recent study further highlighted a potential implication of POPs in the development of obesity in humans [47]. Numerous toxicological studies have also documented that single environmental pollutant may act as endocrine disruptor [41], [43]. However, no experimental study has documented the metabolic effects associated with the consumption of multiple POPs present in a whole food matrix. This issue is of particular interest since humans are mainly exposed to POPs through intake of animal food products like fatty fish, dairy products, and meat [48]. Here, we provide the first evidence that exposure to various POPs through intake of farmed salmon, one of the most consumed fatty fish worldwide, may participate to metabolic disorders linked to type 2 diabetes and obesity. In addition, it appears that environmental doses of POPs are sufficient to provoke harmful effects because concentrations of 7PCBs in adipose tissue of animals fed farmed salmon fillet were similar to those observed in the general population, whereas DDT concentrations were below the human baseline average levels [49], [50]. These results are in accordance with human studies showing that low and environmental doses of POP exposure may increase the risk of insulin resistance [46], [47].

Altogether, these data strongly suggest that human exposure to environmental pollutants has significantly contributed to the epidemic of metabolic syndrome. Limitation of daily and long-term exposure to POPs may therefore represent a novel and attractive approach to slow down the uncontrolled rise of metabolic diseases.

In summary, this study demonstrates that chronic intake of farmed fatty fish contributes, rather than prevents, to insulin resistance and obesity and that these negative effects are, in part, mediated by the presence of POPs in fatty fish. Whether common food products containing POPs like meat and milk products induce unhealthy effects in similar or other dietary models requires further investigations. In addition, future studies should investigate the metabolic impacts of moderate POP-containing farmed salmon consumption –two-three times per week- over long-term. Overall, our findings may provide novel insights regarding future human and clinical nutrition strategies aimed at preventing or treating type 2 diabetes and obesity.

## Supporting Information

Figure S1 **Fat absorption in animals fed VHF.** Feces were collected during 7 days. (**A**) Fecal output, (**B**) total fat excreted in feces, and (**C**) percentage of fat excreted in feces per week (6–13 mice per group). *p<0.0001 vs. C. **p<0.0001 vs. VHF.
(EPS)

Figure S2 **Hepatic TAG levels.** Concentrations of hepatic TAG were assessed in the different experimental groups (7–10 mice per group for VHF trial and 4–6 mice per group for WD trial) *p<0.03 vs. C. **p<0.01 vs. VHF.
(EPS)

Figure S3 **Fat absorption in animals fed WD.** Feces were collected during 7 days. (**A**) Fecal output, (**B**) total fat excreted in feces, and (**C**) percentage of fat excreted in feces per week. (6–13 mice per group). *p<0.03 vs. WD.
(EPS)

Table S1 **Fatty acid composition of diets.** Concentrations of different fatty acids were analysed in experimental diets. ND, not detected.
(DOC)

Table S2 **Environmental pollutants in diets.** Concentrations of POPs in experimental diets. < LOD, below limit of detection. ND, not detected.
(DOC)

Table S3 **Characteristics of farmed salmon fillets.** Commercial farmed Atlantic salmon fillet and farmed Atlantic salmon fillet with reduced POP concentrations were analyzed for protein, lipid and environmental pollutant levels. < LOD, below limit of detection. ND, not detected.
(DOC)
