# Supplementary material for: Chronic Consumption of Farmed Salmon Containing Persistent Organic Pollutants Causes Insulin Resistance and Obesity in Mice
Source: PLoS One. 2011 Sep 23;6(9):e25170. doi: 10.1371/journal.pone.0025170 (PMC3179488; doi:10.1371/journal.pone.0025170)
Supplement: Table S1 — Fatty acid composition of diets. Concentrations of different fatty acids were analysed in experimental diets. ND, not detected. (DOC) [file pone.0025170.s004.doc]

**Table S1. Fatty acid composition of diets.**

| **Fatty acids**  **(mg/g diet)** | **VHF** | **VHF/S** | **VHF/S_-POPs_** | **WD** | **WD/S** |
| --- | --- | --- | --- | --- | --- |
| **Saturated** |  |  |  |  |  |
| 14:0 | 3.45±0.04 | 8.75±0.10 | 11.07±0.18 | ND | 4.46±0.14 |
| 15:0 | ND | 0.54±0.01 | 0.70±0.02 | ND | 0.30±0.01 |
| 16:0 | 73.53±1.08 | 77.66±0.99 | 80.52±1.24 | 15.00±0.31 | 13.73±0.42 |
| 17:0 | 1.03±0.02 | 1.24±0.01 | 1.26±0.03 | ND | 0.23±0.01 |
| 18:0 | 33.85±0.48 | 35.24±0.49 | 34.15±0.58 | 2.82±0.07 | 3.12±0.10 |
| 20:0 | 1.36±0.05 | 1.28±0.07 | 1.16±0.07 | 0.61±0.02 | 0.36±0.02 |
| 22:0 | 0.67±0.06 | 0.24±0.14 | ND | 0.07±0.04 | 0.07±0.0 |
| 24:0 | 0.51±0.01 | ND | ND | 0.23±0.01 | ND |
| Sum | 114.4±1.72 | 124.96±1.52 | 129.7±2.28 | 18.73±0.36 | 22.27±0.70 |
| **Monosaturated** |  |  |  |  |  |
| 16:1n-7 | 5.30±0.09 | 10.49±0.13 | 13.12±0.25 | 0.14±0.04 | 4.47±0.15 |
| 18:1n-7 | 7.38±0.11 | 10.76±0.19 | 9.75±0.23 | 0.79±0.01 | 3.63±0.13 |
| 20:1n-7 | ND | 0.60±0.03 | 0.61±0.02 | ND | 0.21±0.02 |
| 14:1n-9 | ND | ND | ND | ND | ND |
| 16:1n-9 | 0.84±0.01 | 1.16±0.05 | 1.15±0.04 | ND | 0.23±0.01 |
| 18:1n-9 | 149.15±2.39 | 153.03±2.04 | 128.31±2.76 | 40.22±0.82 | 36.67±1.19 |
| 20:1n-9 | 2.28±0.02 | 8.93±0.11 | 9.96±0.26 | 0.31±0.02 | 5.83±0.19 |
| 22:1n-9 | ND | 0.92±0.03 | 0.84±0.02 | ND | 0.68±0.04 |
| 24:1n-9 | ND | 0.63±0.36 | 0.81±0.33 | ND | 0.60±0.25 |
| 18:1n-11 | ND | 0.38±0.22 | 1.00±0.15 | ND | 0.62±0.03 |
| 20:1n-11 | ND | 0.78±0.01 | 1.17±0.07 | ND | 0.59±0.02 |
| 22:1n-11 | ND | 6.78±0.05 | 11.18±0.25 | ND | 5.51±0.21 |
| Sum | 164.95±2.62 | 194.46±2.98 | 177.9±4.22 | 41.46±0.81 | 59.05±1.87 |
| **Polyunsaturated** |  |  |  |  |  |
| 18:2n-6 | 135.54±2.22 | 83.15±1.01 | 80.39±1.60 | 67.71±1.35 | 12.08±0.36 |
| 20:2n-6 | 1.19±0.05 | 2.32±0.02 | 1.85±0.03 | ND | 0.87±0.03 |
| 20:3n-6 | ND | ND | ND | ND | 0.09±0.06 |
| 20:4n-6 | 0.17±0.17 | 1.27±0.20 | 1.32±0.05 | ND | 0.61±0.12 |
| 22:4n-6 | ND | 0.24±0.14 | 0.19±0.12 | ND | 0.13±0.05 |
| 16:2n-4 | ND | 0.54±0.03 | 0.85±0.01 | ND | 0.43±0.02 |
| 16:3n-3 | 1.73±0.03 | 0.81±0.47 | 0.83±0.34 | ND | ND |
| 16:4n-3 | 0.67±0.01 | 0.96±0.01 | 1.11±0.03 | 0.24±0.01 | 0.50±0.04 |
| 18:3n-3 | 3.20±0.06 | 8.12±0.09 | 3.94±0.10 | 1.14±0.02 | 5.11±0.17 |
| 18:4n-3 | ND | 1.65±0.02 | 2.57±0.06 | ND | 1.36±0.06 |
| 20:3n-3 | ND | 0.35±0.20 | ND | ND | 0.28±0.12 |
| 20:4n-3 | ND | 1.89±0.03 | 1.86±0.04 | ND | 1.57±0.06 |
| 20:5n-3 | ND | 6.87±0.04 | 9.54±0.16 | ND | 5.65±0.21 |
| 21:5n-3 | ND | 0.64±0.37 | 0.52±0.32 | ND | 0.43±0.26 |
| 22:5n-3 | ND | 3.57±0.03 | 3.87±0.08 | ND | 2.85±0.10 |
| 22:6n-3 | ND | 11.37±0.09 | 13.92±0.29 | ND | 9.24±0.34 |
| 24:5n-3 | ND | 0.37±0.12 | 0.71±0.02 | ND | 0.38±0.02 |
| Sum n-6 | 136.9±2.22 | 86.74±0.78 | 83.75±1.50 | 67.71±1.35 | 13.78±0.64 |
| Sum n-3 | 5.60±0.09 | 36.60±0.46 | 38.87±0.77 | 1.38±0.02 | 27.37±1.11 |
| n6/n3 | 24.44 | 2.36 | 2.15 | 49.0 | 0.50 |
| Total dietary fatty acids (identified and non-identified) | 423.14±6.66 | 445.96±5.82 | 435.53±8.74 | 129.38±2.53 | 125.48±4.42 |

ND, not detected.
