# Supplementary material for: Chronic Consumption of Farmed Salmon Containing Persistent Organic Pollutants Causes Insulin Resistance and Obesity in Mice
Source: PLoS One. 2011 Sep 23;6(9):e25170. doi: 10.1371/journal.pone.0025170 (PMC3179488; doi:10.1371/journal.pone.0025170)
Supplement: Table S2 — Environmental pollutants in diets. Concentrations of POPs in experimental diets. < LOD, below limit of detection. ND, not detected. (DOC) [file pone.0025170.s005.doc]

**Table S2. Environmental pollutants in diets.**

| **Environmental contaminants (weight/g diet)** | **VHF** | **VHF/S** | **VHF/S_-POPs_** | **WD** | **WD/S** |
| --- | --- | --- | --- | --- | --- |
|  |  |  |  |  |  |
| **Organochlorine pesticides, ng/g** |  |  |  |  |  |
| Alpha-hexachlorocyclohexane | <LOQ | <LOQ | <LOQ | <LOQ | <LOQ |
| Dieldrin | < LOQ | 1.58±0.22 | 0.60±0.01 | < LOQ | 1.21±0.05 |
| Isodrin | < LOQ | < LOQ | < LOQ | < LOQ | < LOQ |
| Heptachlor | <LOQ | <LOQ | <LOQ | <LOQ | <LOQ |
| Aldrin | < LOQ | < LOQ | < LOQ | < LOQ | < LOQ |
| Oxychlordane | < LOQ | < LOQ | < LOQ | < LOQ | < LOQ |
| Heptachlor A | < LOQ | < LOQ | < LOQ | < LOQ | < LOQ |
| Trans-chlordane | < LOQ | < LOQ | < LOQ | < LOQ | < LOQ |
| Alpha-endosulfan | < LOQ | < LOQ | < LOQ | < LOQ | < LOQ |
| Cis-chlordane | < LOQ | < LOQ | < LOQ | < LOQ | < LOQ |
| Trans-nonachlor | < LOQ | 0.84±0.08 | 0.32±0.02 | < LOQ | 0.64±0.06 |
| Toxaphene-26 | < LOQ | < LOQ | < LOQ | < LOQ | < LOQ |
| Beta-endosulfan | < LOQ | < LOQ | < LOQ | < LOQ | < LOQ |
| Cis-nonachlor | < LOQ | 0.39±0.04 | < LOQ | < LOQ | < LOQ |
| Toxaphene-32 | < LOQ | < LOQ | < LOQ | < LOQ | < LOQ |
| Endosulfan-sulfate | < LOQ | 0.34±0.02 | < LOQ | < LOQ | < LOQ |
| Toxaphene-50 | < LOQ | 1.19±0.13 | < LOQ | < LOQ | < LOQ |
| Toxaphene-62 | < LOQ | < LOQ | < LOQ | < LOQ | < LOQ |
| Toxaphene-40+41 | < LOQ | < LOQ | < LOQ | < LOQ | < LOQ |
| Mirex | < LOQ | < LOQ | < LOQ | < LOQ | < LOQ |
| Hexachlorobenzene | < LOQ | 1.07±0.11 | < LOQ | < LOQ | < LOQ |
| *Sum* | *< LOQ* | *5.42* | *0.92* | *< LOQ* | *1.85* |
| **Dichloro-Diphenyl-Trichloroethanes (DDTs), ng/g** |  |  |  |  |  |
| op’-DDT | < LOQ | < LOQ | < LOQ | < LOQ | < LOQ |
| pp’-DDT | < LOQ | < LOQ | < LOQ | < LOQ | < LOQ |
| op’-DDD | < LOQ | < LOQ | < LOQ | < LOQ | < LOQ |
| pp’-DDD | < LOQ | 1.99±0.35 | 1.05±0.08 | < LOQ | 1.21±0.04 |
| op’-DDE | < LOQ | < LOQ | < LOQ | < LOQ | < LOQ |
| pp’-DDE | < LOQ | 4.44±0.60 | 1.70±0.12 | < LOQ | 3.59±0.18 |
| *Sum* | *< LOQ* | *6.42* | *2.75* | *< LOQ* | *4.80* |
| **Dioxins (PCDDs, PCDFs and dioxin-like PCBs):** |  |  |  |  |  |
| **Polychlorinated dibenzo-p-dioxins (PCDDs), pg/g** |  |  |  |  |  |
| 2,3,7,8-Tetrachlorodibenzo-p-dioxin (2,3,7,8-TCDD) | 0.0054±0.002 | 0.0324±0.009 | 0.0325±0.014 | 0.0063±0.005 | 0.0163±0.002 |
| 1,2,3,7,8-Pentachlordibenzodioxin (1,2,3,7,8-PeCDD) | 0.0147±0.012 | 0.0554±0.027 | 0.0097±0.003 | ND | 0.0132±0.007 |
| 1,2,3,4,7,8-Hexachlordibenzodioxin (1,2,3,4,7,8-HxCDD) | 0.0124±0.003 | 0.0149±0.002 | 0.0101±0.002 | 0.0048±0.003 | 0.0093±0.005 |
| 1,2,3,6,7,8-Hexachlordibenzodioxin (1,2,3,6,7,8-HxCDD) | 0.0326±0.015 | 0.0750±0.022 | 0.0291±0.001 | 0.0069±0.002 | 0.0452±0.025 |
| 1,2,3,7,8,9-Hexachlordibenzodioxin (1,2,3,7,8,9-HxCDD) | 0.0091±0.0002 | 0.0173±0.009 | 0.0150±0.003 | 0.0009±0.001 | 0.0044±0.004 |
| 1,2,3,4,6,7,8-Heptachlorodibenzo-p-dioxin (1,2,3,4,6,7,8-HpCDD) | 0.1612±0.007 | 0.1895±0.015 | 0.1837±0.029 | 0.0221±0.004 | 0.0128±0.006 |
| Octachlorodibenzodioxin (OCDD) | 1.2574±0.029 | 1.5137±0.025 | 1.3721±0.032 | 0.3997±0.020 | 0.0432±0.013 |
| *Sum* | *1.4928* | *1.8982* | *1.6522* | *0.4442* | *0.1409* |
| **Polychlorinated dibenzofurans (PCDFs), pg/g** |  |  |  |  |  |
| 2,3,7,8-Tetrachlorodibenzofuran (2,3,7,8-TCDF) | 0.0794±0.001 | 0.4689±0.003 | 0.3468±0.085 | 0.0952±0.015 | 0.4332±0.20 |
| 1,2,3,7,8-Pentachlorodibenzofuran (1,2,3,7,8-PeCDF) | 0.0044±0.004 | 0.0426±0.020 | 0.0151±0.005 | ND | 0.0459±0.012 |
| 2,3,4,7,8-Pentachlorodibenzofuran (2,3,4,7,8-PeCDF) | 0.0043±0.004 | 0.1355±0.001 | 0.0690±0.008 | 0.0007±0.001 | 0.1096±0.022 |
| 1,2,3,4,7,8-Hexachlorodibenzofuran (1,2,3,4,7,8-HxCDF) | 0.0012±0.001 | 0.0128±0.004 | 0.0064±0.006 | 0.0001±0.0001 | ND |
| 1,2,3,6,7,8-Hexachlorodibenzofuran (1,2,3,6,7,8-HxCDF) | 0.0080±0.002 | 0.0290±0.012 | ND | ND | 0.0033±0.002 |
| 1,2,3,7,8,9-Hexachlorodibenzofuran (1,2,3,7,8,9-HxCDF) | ND | ND | ND | ND | ND |
| 2,3,4,6,7,8-Hexachlorodibenzofuran (2,3,4,6,7,8-HxCDF) | 0.0123±0.008 | 0.0404±0.013 | 0.0262±0.001 | 0.0071±0.00001 | 0.0241±0.005 |
| 1,2,3,4,6,7,8-Heptachlorodibenzofuran (1,2,3,4,6,7,8-HpCDF) | 0.0268±0.010 | 0.0620±0.013 | 0.0577±0.004 | 0.0039±0.001 | 0.0120±0.009 |
| 1,2,3,4,7,8,9-Heptachlorodibenzofuran (1,2,3,4,7,8,9-HpCDF) | 0.0028±0.003 | 0.0177±0.018 | 0.0076±0.008 | ND | 0,0026±0.001 |
| Octachlorodibenzofuran (OCDF) | 0.0088±0.009 | 0.0337±0.034 | 0.0133±0.013 | ND | ND |
| *Sum* | *0.1480* | *0.8426* | *0.5421* | *0.1070* | *0.6307* |
| **Non-ortho-substituted PCBs, pg/g** |  |  |  |  |  |
| 3,3',4,4'- Tetrachlorobiphenyl (CB-77) | 0.514±0.03 | 12.274±0.36 | 9.172±0.59 | 0.520±0.04 | 10.832±0.86 |
| 3,4,4',5- Tetrachlorobiphenyl (CB-81) | 0.042±0.01 | 0.529±0.02 | 0.380±0.04 | 0.015±0.002 | 0.395±0.08 |
| 3,3',4,4',5- Pentachlorobiphenyl (CB-126) | 0.084±0.01 | 3.156±0.16 | 2.017±0.21 | 0.054±0.01 | 2.832±0.16 |
| 3,3',4,4',5,5'- Hexachlorobiphenyl (CB-169) | 0.024±0.0004 | 0.526±0.01 | 0.463±0.02 | 0.007±0.002 | 0.448±0.02 |
| *Sum* | *0.66* | *16.48* | *12.03* | *0.60* | *14.51* |
| ***Mono-ortho-substituted PCBs,* pg/g** |  |  |  |  |  |
| 2,3,3,'4,4'- Pentachlorobiphenyl (CB-105) | ND | 195.571±2.30 | 107.294±0.93 | ND | 169.924±11.41 |
| 2,3,4,4',5- Pentachlorobiphenyl (CB 114) | 0.158±0.003 | 12.523±0.61 | 5.818±0.38 | ND | 10.656±0.79 |
| 2,3',4,4',5- Pentachlorobiphenyl (CB-118) | ND | 683.008±7.12 | 340.735±1.09 | ND | 586.522±24.52 |
| 2',3,4,4',5- Pentachlorobiphenyl (CB-123) | 0.125±0.03 | 17.613±1.92 | 7.592±0.61 | 0.213±0.19 | 17.983±2.11 |
| 2,3,3',4,4',5- Hexachlorobiphenyl (CB-156) | ND | 58.181±1.08 | 37.881±0.24 | ND | 50.729±2.25 |
| 2,3,3',4,4',5'- Hexachlorobiphenyl (CB-157) | 0.114±0.09 | 17.277±1.69 | 11.974±0.16 | ND | 13.538±0.32 |
| 2,3',4,4',5,5'- Hexachlorobiphenyl (CB-167) | 0.234±0.17 | 39.915±0.02 | 25.397±0.28 | 0.104±0.10 | 35.927±0.82 |
| 2,3,3',4,4',5,5'- Heptachlorobiphenyl (CB-189) | 0.168±0.04 | 7.345±0.19 | 5.461±0.003 | 0.060±0.05 | 5.5630±0.49 |
| *Sum* | *0.80* | *1031.43* | *542.15* | *0.38* | *890.84* |
| *Total dioxins* | *3.10* | *1050.66* | *556.38* | *1.52* | *906.12* |
| **Polychlorinated biphenyls (PCBs), ng/g** |  |  |  |  |  |
| 2,4,4'-Trichlorobiphenyl (CB-28) | < LOQ | < LOQ | < LOQ | < LOQ | < LOQ |
| 2,2',5,5'-Tetrachlorobiphenyl (CB-52) | < LOQ | 0.400±0.06 | 0.195±0.01 | < LOQ | 0.315±0.01 |
| 2,2',4,5,5'-Pentachlorobiphenyl (CB-101) | < LOQ | 0.935±0.00 | 0.435±0.01 | < LOQ | 0.835±0.06 |
| 2,3',4,4',5-Pentachlorobiphenyl (CB-118) | < LOQ | 0.695±0.01 | 0.365±0.01 | < LOQ | 0.605±0.05 |
| 2,2',3,4,4',5'-Hexachlorobiphenyl (CB-138) | < LOQ | 1.600±0.00 | 0.955±0.02 | < LOQ | 1.400±0.10 |
| 2,2',4,4',5,5'-Hexachlorobiphenyl (CB-153) | < LOQ | 1.600±0.00 | 0.885±0.01 | < LOQ | 1.500±0.10 |
| 2,2',3,4,4',5,5'-Heptachlorobiphenyl (CB-180) | < LOQ | 0.435±0.01 | 0.320±0.00 | < LOQ | 0.405±0.03 |
| *Sum ICES7* | *< LOQ* | *5.665* | *3.155* | *< LOQ* | *5.060* |
