# Supplementary material for: Chronic Consumption of Farmed Salmon Containing Persistent Organic Pollutants Causes Insulin Resistance and Obesity in Mice
Source: PLoS One. 2011 Sep 23;6(9):e25170. doi: 10.1371/journal.pone.0025170 (PMC3179488; doi:10.1371/journal.pone.0025170)
Supplement: Table S3 — Characteristics of farmed salmon fillets. Commercial farmed Atlantic salmon fillet and farmed Atlantic salmon fillet with reduced POP concentrations were analyzed for protein, lipid and environmental pollutant levels. < LOD, below limit of detection. ND, not detected. (DOC) [file pone.0025170.s006.doc]

**Table S3. Characteristics of farmed salmon fillets.**

|  | **Commercial farmed Atlantic salmon fillet** | **Farmed Atlantic salmon fillet with reduced POP concentrations** |
| --- | --- | --- |
|  |  |  |
| ***Crude protein (g/100g dry weight):*** | 59.7±0.6 | 59.3±0.4 |
| ***Lipid content (g/100g dry weight):*** | 38.8±0.1 | 36.8±0.6 |
| ***Environmental pollutants:*** |  |  |
| **PCBs (µg/kg wet weight):** |  |  |
| CB-28 | <LOD | <LOD |
| CB-52 | 0.34±0.02 | 0.12±0.01 |
| CB-101 | 0.66±0.04 | 0.27±0.01 |
| CB-118 | 0.39±0.02 | 0.21±0.01 |
| CB-138 | 0.71±0.04 | 0.47±0.04 |
| CB-153 | 0.77±0.04 | 0.45±0.03 |
| CB-180 | <LOD | <LOD |
| 7 PCBs | 2.88±0.15 | 1.53±0.11 |
| **DDTs (µg/kg wet weight)** |  |  |
| op'-DDT | <LOD | <LOD |
| pp'-DDT | 0.38±0.02 | <LOD |
| op'-DDD | <LOD | <LOD |
| pp'-DDD | 0.85±0.06 | 0.44±0.04 |
| op'-DDE | <LOD | <LOD |
| pp'-DDE | 2.10±0.12 | 0.96±0.03 |
| Sum | 3.34±0.19 | 1.40±0.07 |
| ***Amino acids (mg/g wet weight):*** |  |  |
| Histidine | 4.58±0.06 | 4.57±0.26 |
| Taurine | 0.31±0.01 | 0.47±0.01 |
| Serine | 6.66±0.05 | 6.77±0.22 |
| Arginine | 9.38±0.13 | 9.85±0.30 |
| Glycine | 7.64±0.11 | 8.17±0.15 |
| Aspartic acid | 17.64±0.24 | 17.37±0.01 |
| Glutamic acid | 22.77±0.26 | 22.08±0.33 |
| Threonine | 8.02±0.05 | 8.14±0.28 |
| Alanine | 10.46±0.07 | 10.16±0.09 |
| Proline | 5.20±0.02 | 5.84±0.12 |
| Lysine | 16.21±0.31 | 14.94±0.16 |
| Tyrosine | 5.76±0.09 | 5.88±0.45 |
| Methionine | 5.19±0.05 | 5.22±0.23 |
| Valine | 9.22±0.03 | 8.95±0.25 |
| Isoleucine | 7.97±0.03 | 7.85±0.20 |
| Leucine | 13.32±0.07 | 13.24±0.29 |
| Phenylalanine | 6.85±0.11 | 7.57±0.39 |
| ***Fatty acids (mg/g wet weight):*** |  |  |
| **Saturated** |  |  |
| 14:0 | 4.02±0.08 | 5.78±0.20 |
| 15:0 | 0.30±0.01 | 0.38±0.01 |
| 16:0 | 12.17±0.23 | 14.64±0.49 |
| 17:0 | 0.21±0.001 | 0.24±0.01 |
| 18:0 | 2.77±0.05 | 2.78±0.08 |
| 20:0 | 0.26±0.002 | 0.27±0.01 |
| 22:0 | 0.07±0.04 | ND |
| 24:0 | ND | ND |
| **Monounsaturated** |  |  |
| 16:1n-7 | 3.94±0.08 | 6.04±0.18 |
| 18:1n-7 | 3.28±0.06 | 2.57±0.08 |
| 20:1n-7 | 0.19±0.004 | 0.21±0.004 |
| 14:1n-9 | ND | ND |
| 16:1n-9 | 0.25±0.02 | 0.21±0.004 |
| 18:1n-9 | 32.46±0.67 | 13.14±0.41 |
| 20:1n-9 | 5.45±0.10 | 6.19±0.23 |
| 22:1n-9 | 0.66±0.01 | 0.59±0.03 |
| 24:1n-9 | ND | 0.51±0.02 |
| 18:1n-11 | 0.54±0.02 | 1.10±0.04 |
| 20:1n-11 | 0.57±0.01 | 0.89±0.04 |
| 22:1n-11 | 5.31±0.11 | 8.52±0.31 |
| **Polyunsaturated** |  |  |
| 18:2n-6 | 10.47±0.22 | 5.39±0.16 |
| 20:2n-6 | 0.80±0.02 | 0.47±0.02 |
| 20:3n-6 | 0.20±0.01 | 0.18±0.001 |
| 20:4n-6 | 0.36±0.02 | 0.54±0.002 |
| 16:2n-4 | 0.38±0.01 | 0.63±0.02 |
| 16:3n-3 | ND | 0.46±0.01 |
| 16:4n-3 | 0.41±0.03 | 0.57±0.04 |
| 18:3n-3 | 4.56±0.10 | 1.42±0.05 |
| 18:4n-3 | 1.26±0.03 | 1.97±0.07 |
| 20:3n-3 | 0.45±0.02 | 0.15±0.03 |
| 20:4n-3 | 1.42±0.03 | 1.39±0.05 |
| 20:5n-3 | 5.20±0.10 | 7.24±0.20 |
| 22:5n-3 | 2.62±0.05 | 2.87±0.09 |
| 22:6n-3 | 8.65±0.01 | 10.71±0.31 |
